# Bap and Cell Surface Hydrophobicity Are Important Factors in *Staphylococcus xylosus* Biofilm Formation

**DOI:** 10.3389/fmicb.2019.01387

**Published:** 2019-06-25

**Authors:** Carolin Schiffer, Maik Hilgarth, Matthias Ehrmann, Rudi F. Vogel

**Affiliations:** Lehrstuhl für Technische Mikrobiologie, Technische Universität München, Freising, Germany

**Keywords:** *Staphylococcus xylosus*, biofilm, biofilm associated protein (Bap), surface hydrophobicity, coagulase negative staphylococci

## Abstract

*Staphylococcus (S.) xylosus* is a coagulase-negative *Staphylococcus* species naturally present in food of animal origin with a previously described potential for biofilm formation. In this study we characterized biofilm formation of five selected strains isolated from raw fermented dry sausages, upon different growth conditions. Four strains exhibited a biofilm positive phenotype with strain-dependent intensities. Biofilm formation of *S. xylosus* was influenced by the addition of glucose, sodium chloride and lactate to the growth medium, respectively. It was further dependent on strain-specific cell surface properties. Three strains exhibited hydrophobic and two hydrophilic cell surface properties. The biofilm positive hydrophilic strain TMW 2.1523 adhered significantly better to hydrophilic than to hydrophobic supports, whereas the differences in adherence to hydrophobic versus hydrophilic supports were not as distinct for the hydrophobic strains TMW 2.1023, TMW 2.1323, and TMW 2.1521. Comparative genomics enabled prediction of functional biofilm-related genes and link these to phenotypic variations. While a wide range of biofilm associated factors/genes previously described for *S. aureus* and *S. epidermidis* were absent in the genomes of the five strains analyzed, they all possess the gene encoding biofilm associated protein Bap. The only biofilm negative strain TMW 2.1602 showed a mutation in the *bap* sequence. This study demonstrates that Bap and surface hydrophobicity are important factors in *S. xylosus* biofilm formation with potential impact on the assertiveness of a starter strain against autochthonous staphylococci by competitive exclusion during raw sausage fermentation.

## Introduction

*Staphylococcus (S.) xylosus* is a Gram-positive, coagulase negative species often found on mammal skin. *S. xylosus* is also widely used as starter organism in raw sausage fermentations (Vos et al., 2009) and has been described as biofilm producer in the past (Planchon et al., 2006; Xu et al., 2017). This ability can be positively associated with food fermentation processes, as adhesion and biofilm formation may increase the assertiveness of a starter organism against the autochthonous microbiota by concomitant induction of colonization resistance in a particular ecological niche. Additionally, biofilms offer a physical protection to bacteria against stress factors including antimicrobial substances (An and Friedman, 2010). In general, the lifecycle of a biofilm can be divided into the stages attachment, maturation and detachment (Otto, 2008). Thereby the first two stages are the main steps of the biofilm formation process, in which multiple factors are involved, and which is often dependent on environmental factors and availability of nutrients (Götz, 2002). Primarily, adherence to a certain support is mediated by nonspecific and/or specific adhesion factors. The latter are termed microbial surface components recognizing adhesive matrix molecules (MSCRAMMS), comprising adhesins on the cell surface of bacteria that bind specifically to extracellular matrix proteins, such as collagen, fibronectin or elastin (An and Friedman, 2010). Following initial adhesion, biofilm accumulation sets in with cells adhering to each other and producing a matrix in which they are embedded in. This extracellular matrix is usually composed of polysaccharides, proteins, and eDNA (Flemming and Wingender, 2010). The multifactorial mechanisms involved in biofilm formation of staphylococci have been described extensively for *S. aureus* and *S. epidermidis* in the past (Götz, 2002; Fey and Olson, 2010), often focusing on two important gene loci with functional redundancy, i.e., presence of either one correlates with strong biofilm production (Moretro et al., 2003; Cucarella et al., 2004; Tormo et al., 2005). The polysaccharide intercellular adhesin (PIA), which is synthesized by the products of the *ica* operon (Cramton et al., 1999) and the biofilm associated protein (Bap). Members of the Bap family are known to be involved in adhesion and biofilm forming processes (Latasa et al., 2006) and comprise among others Bhp, a surface protein often found in *S. epidermidis* (Tormo et al., 2005) and Esp, a surface protein found in *Enterococcus faecalis* (Shankar et al., 1999).

This study aimed to characterize phenotypic variations among different strains of *S. xylosus* regarding their ability to form biofilms, investigate factors influencing biofilm formation, and employed comparative genomic analysis to further comprehend primary adhesion and biofilm accumulation mechanisms in *S. xylosus.*

## Materials and Methods

### Bacterial Strains and Culture Conditions

Five *S. xylosus* strains from the strain collection of Technische Mikrobiologie Weihenstephan (TMW), which were originally isolated from raw fermented sausages, and *S. epidermidis* RP62A obtained from DSMZ were selected for all experiments. Unless otherwise indicated, strains were grown from cryocultures in tryptic soy broth (TSB, casein peptone 15 g/l, soy peptone 15 g/l, yeast extract 3 g/l) aerobically cultivated until stationary phase (approximately 18 h) at 37°C and shaken at 200 rpm until further use.

### Congo Red Agar Assay

To screen for slime production, the congo red agar test was performed as described by Freeman et al. (1989). Briefly, cultures were cultivated on a mixture of 37 g/l brain heart infusion broth (Carl Roth, Germany), 10 g/l agar and 50 g/l sucrose. The medium was supplemented with a solution of separately autoclaved 0.8 g/l of Congo Red (Carl Roth, Germany). After incubation of the isolates on the plates for 24 h at 37°C and 12 h at room temperature, plates were screened for differences in colony morphology. Black and dry crystalline colonies reveal slime producer, while non-slime producer usually develop pink and smooth colonies. Pictorial examples for different kinds of phenotypes is given in Knobloch et al. (2002).

### Quantitative Biofilm Formation Assay on Hydrophilic and Hydrophobic Support in Different Cultivation Media

Biofilm formation was tested according to Christensen et al. (1985), with some minor modifications. Basically, overnight cultures of the selected strains were washed and diluted to an OD_590_ of 0.05 in medium. 200 μl of the adjusted cultures were pipetted into the wells of a 96-well plate and statically incubated for 24 h. After incubation, OD_590_ was measured again to confirm adequate cell growth in all wells. The wells were carefully decanted and plates were washed twice with sterile phosphate buffered saline (PBS) (NaCl 9 g/l, Na_2_HPO_4_^∗^7H_2_O 0.795 g/l, KH_2_PO_4_ 0.114 g/l, pH 7.2). For biofilm fixation, plates were dried in an inverted position in a heat chamber (60°C) for at least 1 h. Adherent biofilm was stained with 200 μl 0.1% safranin-O (Sigma Aldrich, United States) for 5 min. Unbound safranin was removed, and plates were washed again twice with PBS. After air drying of the plates, the stain was solubilized with ethanol (95%) and absorbance was quantified at 490 nm.

In order to test dependence of phenotypic variations and expression of a biofilm positive phenotype on the presence of certain substances, the biofilm assay was performed using different cultivation media (TSB, TSB + 1% glucose, TSB + 1% glucose + 3% sodium chloride, using lactic acid). Additionally, two different supports were used, polystyrene 96-well plates (Sarstedt, Germany) and Nunclon^TM^ delta surface 96-well plates (Thermo Fisher Scientific, United States) as hydrophobic and hydrophilic representatives, respectively.

Experiments were conducted in at least three independent biological replicates. Each biological replicate was performed in technical triplicates. Wells containing sterile medium only, served as a control in every experiment performed. *S. epidermidis* RP62A described as a strong biofilm producer and commonly used as model strain (Mack et al., 1992; Conlon et al., 2002) was included as a positive control for biofilm formation into the experiments.

### Microbial Adhesion to Hydrocarbon (MATH)

For determining the surface hydrophobicity of cells, the adherence of bacteria to *n*-hexadecane was measured as described by Rosenberg (2006). Cells from overnight cultures were washed and resuspended in imidazole/PBS (KH_2_PO_4_ 0.1 g/l, Na_2_HPO_4_^∗^2H_2_O 4.45 g/l, imidazole 1.7 g/l, pH 6.2) to an OD_590_ of 0.35 to 0.4 (A_B_). 5 ml of the cell suspension were overlaid with 0.4 ml *n*-hexadecane (Sigma Aldrich, United States) and incubated for 10 min at 37°C. Mixtures were then vortexted for 2 min and statically incubated for another 15 min at room temperature until phase separation was completed. The absorbance (A_A_) of the aqueous phase was measured and the affinity for *n*-hexadecane (%) determined by using the following formula:

$$affinity\;to\;n\mathrm{-hexadecane}\;(\%)=\frac{A_{B}-A_{A}}{A_{B}}x\;100$$

If values were over 50%, strains were considered as highly hydrophobic, if values were under 20%, as hydrophilic. Each experiment was conducted in three independent runs.

### DNA Isolation, Sequencing and Bioinformatics Analysis

For isolation of high-molecular-weight DNA from liquid (tryptic soy broth) bacterial overnight cultures, the E.Z.N.A^®^kit (Omega Bio-Tek Inc., United States) was used. Whole genome sequencing followed using SMRT (Single molecule real time) sequencing technology (PacBio RS II). The sequencing was carried out at GATC Biotech (Konstanz, Germany). For library creation an insert size of 8 to 12 kb was constructed, delivering at least 200 Mb of raw data from one to two SMRT cells (1 × 120-min movies), when P4-C2 chemistry is applied. SMRT Analysis version 2.2.0.p2 and the hierarchical genome assembly process (HGAP) were used for *de novo* assembly (Chin et al., 2013). Completion by manual processing according to PacBio instructions followed. Annotation of the genomes was based on the NCBI Prokaryotic Genome Annotation Pipeline (PGAP) and the Rapid Annotations using Subsystems Technology (RAST) Server (Aziz et al., 2008; Tatusova et al., 2016). Bioinformatic analysis and comparative genomics were performed using CLC Main Workbench 8 software (CLC bio, Denmark). To determine strain diversity, average nucleotide identity (ANI) values were calculated using additionally available whole genome sequencing data of four other *S. xylosus* strains (C2A (LN554884), S170 (CP013922), HKUOPL8 (CP007208), and SMQ-121 (CP008724)). Therefore, the ANIb algorithm (Goris et al., 2007) which is implemented within JspeciesWS web service (Richter et al., 2016) was applied and a neighbor-joining distance tree was built using MEGA7 software.

### Statistical Analysis

For statistical analysis, Shapiro–Wilk test was performed to assure normal distribution of data. Means of the technical triplicates were determined first, followed by calculating the means of the biological triplicates including error propagation, which were then used for subsequent statistical comparison of differences. Two-tailed Student’s *t*-tests assuming unequal variances were performed using SigmaPlot Version 12.5 (Systat Software GmbH, Germany). A difference of means was considered as being significant if *p*-values were less than 0.05 (*P* < 0.05). Student’s *t*-test were performed to compare biofilm intensities of the strains on hydrophilic vs. hydrophobic support and in TSB supplemented with glucose compared to TSB, TSB supplemented with 3% NaCl + 1% glucose compared to TSB + 1% glucose as well as TSB + 1% glucose + lactate (pH 6) compared to TSB + 1% glucose.

## Results

### Surface Hydrophobicity

According to the MATH test, only two of the tested strains possess hydrophilic surface properties (TMW 2.1523, TMW 2.1602). All other strains expressed a decisive affinity for the hydrocarbon phase, thus can be considered as strongly hydrophobic (Table 1).

**Table 1:** Surface hydrophobicity of *S. xylosus* TMW strains and *S. epidermidis* RP62A.

Strain	Affinity for *n*-hexadecane (%)	Degree of Hydrophobicity
*S. xylosus* TMW 2.1023	95.0 ± 0.2	strong
*S. xylosus* TMW 2.1324	89.7 ± 3.1	strong
*S. xylosus* TMW 2.1521	93.4 ± 2.7	strong
*S. xylosus* TMW 2.1523	0.6 ± 1.1	weak
*S. xylosus* TMW 2.1602	0.9 ± 1.9	weak
*S. epidermidis* RP62A	95.6 ± 1.7	strong


*Mean ± SE.*

### Behavior of Colonies in the Congo Red Agar Assay

All *S. xylosus* isolates were tested negative for slime production by the congo red agar test. Colonies were mostly smooth, shiny and pink. Yet, changes to a darker color in parts where colonies were in close proximity to each other were observed for TMW 2.1523. The colonies of TMW 2.1523 also showed a rough instead of a smooth surface and a lobate margin. A dry surface with a lobate margin was observed for TMW 2.1521 as well. However, the typical overall black and dry crystalline morphology of a slime producer couldn’t be detected for any of the *S. xylosus* strains. *S. epidermidis* RP62A served as positive control.

### Influence of Support Hydrophobicity on Biofilm Formation

**FIGURE 1:**
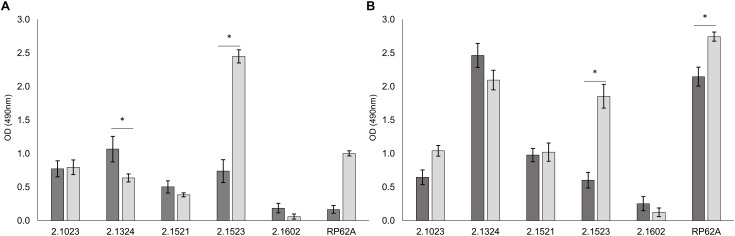
Biofilm formation is dependent on surface hydrophobicity of the support. Biofilm formation on hydrophobic (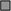) and hydrophilic (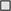) support in TSB **(A)** and TSB + 1% glucose **(B)**. Significant differences of mean are marked by ^∗^. Mean ± SE.

Adherence potential of *S. xylosus* to either hydrophobic or hydrophilic supports differed as shown in Figure 1. Among the strains that proved to be of hydrophobic nature, TMW 2.1023 and TMW 2.1521 weakly (A_490_ < 1.5) adhered to both supports, TMW 2.1324 adhered slightly better to hydrophobic than to hydrophilic support and *S. epidermidis* RP62A formed significantly more biofilm on hydrophilic than on hydrophobic support. Among the two hydrophilic strains, TMW 2.1602 adhered to neither of the supports (A_490_< 0.5), while TMW 2.1523 produced significantly more biofilm on hydrophilic compared to the hydrophobic support. In general, relations of biofilm formation on the two tested supports were similar in TSB and TSB + 1% glucose (compare Figure 1A,B), implicating that medium composition had no major influence on the adherence preference of the examined strains to either of the supports. Moreover, *S. xylosus* proved to be able to form comparable intensities of biofilm as the well characterized biofilm producer *S. epidermidis* RP62A.

### Influence of Media Composition on Biofilm Formation

**FIGURE 2:**
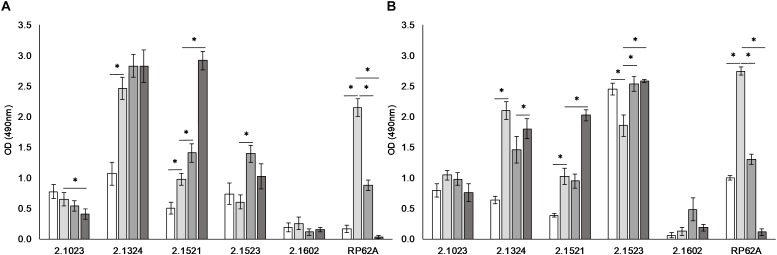
Biofilm formation is dependent on environmental conditions. Biofilm formation on hydrophobic **(A)** and hydrophilic **(B)** support when incubated in TSB (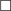), TSB + 1% glucose (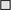), TSB + 1% glucose + 3% NaCl (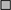), and TSB + 1% glucose + lactate (pH 6) (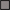). Significant differences of mean are marked by ^∗^. Mean ± SE.

Media composition was found to influence adherence potential in a strain dependent matter (Figure 2). *S. xylosus* strain TMW 2.1602 proved again to be a non-biofilm producer regardless of which additive the media contained (A_490_ < 0.5).

*S. xylosus* TMW strains 2.1324 and 2.1521 as well as *S. epidermidis* RP62A displayed significantly enhanced biofilm formation on both supports tested upon the addition of 1% glucose to the culture medium. On the contrary, biofilm formation was significantly reduced by the presence of glucose in TMW 2.1523 on hydrophilic support. In weak biofilm producer TMW 2.1023, supplementation of glucose had no significant effect on adherence potential. Upon the addition of 3% NaCl to the culture medium, no clear pattern was identifiable for TMW strains 2.1023, 2.1324, and 2.1521. However, biofilm formation was significantly enhanced with NaCl present in TMW 2.1523 and significantly reduced in *S. epidermidis* RP62A on both supports, respectively. Acidification to pH 6 by lactate had a significantly enhancing effect on biofilm formation of TMW 2.1521 and 2.1523 while it significantly reduced biofilm formation of RP62A. The promoting effect of lactate on biofilm formation was especially distinct in TMW 2.1521, as the strain displayed a weak biofilm phenotype in TSB, TSB enriched with glucose and TSB enriched with a combination of glucose and NaCl. Using lactic acid, however, enhanced the strains biofilm formation to a degree that was comparable to the strong biofilm formers *S. xylosus* TMW 2.1324, 2.1523 and *S. epidermidis* RP62A (A_490_ > 2.0).

### General Genome Features

Supplementary Table S1 summarizes the main genome features of the sequenced strains as well as the respective accession numbers. All five sequenced *S. xylosus* strains possess a single circular chromosome with sizes ranging from 2.8 to 2.9 Mbp, a GC content of 32.7 – 32.9 mol% and a strain-dependent plasmid quantity. The calculated ANI values (Supplementary Figure S1) confirmed genomic diversity among the isolates and revealed certain groups within the species *S. xylosus*. One comprising most of the TMW strains as well as *S. xylosus* C2A, originating from human skin (Götz et al., 1983) and *S. xylosus* SMQ-121, a starter used in the fermentation of processed meat (Labrie et al., 2014). Within this group, the strains TMW 2.1023 and TMW 2.1521 show the lowest genomic distance, while TMW 2.1523 seems to be considerably different from all the other *S. xylosus* strains. The second group comprised TMW 2.1602 and two additional *S. xylosus* strains that were both isolated in Asia, S170 from leaf vegetables (Hong and Roh, 2018) and HKUOPL8 from feces of healthy panda (Ma et al., 2014).

### Genetic Screening for Adhesion and Biofilm Formation Related Factors

To further investigate the observed phenotypic differences, sequenced genomes of the five *S. xylosus* isolates were screened for the presence of genes that have been described to be associated with adhesion and biofilm formation processes of well characterized biofilm producers *S. epidermidis* and *S. aureus* (Table 2). *S. xylosus* carries only a small fraction of the described genes, among them autolysin *atl*/*atlE*, known to be involved in unspecific adhesion, MCSCRAMMs such as *ebpS, eno, fnb* as well as *bap*, a protein important in the biofilm accumulation process. Other genes, also associated with biofilm accumulation, such as *aap* and the *ica*-operon are lacking in all *S. xylosus* strains. Solely TMW 2.1602 carries parts of the *ica* operon, however, *icaD* is missing and only *icaR, icaC, icaB*, and *icaA* are present in the genome. Moreover, six out of eight genes of the *ess* cluster, encoding the ESAT-6 secretion system (ESS), were detected in TMW 2.1523 (*esxA, esaA, essA, esaB, essB, essC*, and A2I72_12780-12805). Compared to the *ess* cluster of *S. aureus* Newman (Burts et al., 2008), only *esaC* and *esxB* are missing in TMW 2.1523, both of which encode secreted polypeptides. All biofilm related genes analyzed in this study are located on the chromosome of the corresponding *S. xylosus* strains and not on their plasmids.

**Table 2:** Analysis of adhesion and biofilm associated genes, described for *S. aureus* and *S. epidermidis* regarding their presence in the sequenced genomes of *S. xylosus* TMW 2.1023, TMW 2.1324, TMW 2.1521, TMW 2.1523, and TMW 2.1602.

Gene	Product	*S. aureus*	*S. epidermidis*	2.1023	2.1324	2.1521	2.1523	2.1602
*aap*/*sas*G	Accumulation associated protein	Corrigan et al., 2007	Schaeffer et al., 2015	–	–	–	–	–
*atl*/*atl*E	Autolysin	Bose et al., 2012	Heilmann et al., 1997	A2I69_04060	A2I70_04320	A2I71_09220	A2I72_04315	A2I73_09055
*bap*	Biofilm associated protein	Cucarella et al., 2001	Tormo et al., 2005	A2I69_12090	A2I70_12670	A2I71_01190	A2I72_12310	truncated A2I73_01165
*bhp*	Bap homolog protein	–	Tormo et al., 2005	–	–	–	–	–
*clf*A, *clf*B	Clumping factors A and B	McDevitt et al., 1994; Ní Eidhin et al., 1998	–	–	–	–	–	–
*cna*	Collagen adhesion protein	Patti et al., 1992	–	–	–	–	–	–
*eap*/*map*	Extracellular adhesion protein	Jönsson et al., 1995; Palma et al., 1999	–	–	–	–	–	–
*ebh*/*embp*	Extracellular matrix binding protein	Clarke et al., 2002	Williams et al., 2002	–	–	–	–	–
*ebp*S	Elastin binding protein	Downer et al., 2002	–	A2I69_06275	A2I70_06530	A2I71_07005	A2I72_06515	A2I73_06955
*efb* (*fib*	Fibronectin / fibrinogen adhesin	Palma et al., 1998	–	–	–	–	–	–
*eno*	Laminin binding protein	Carneiro et al., 2004	–	A2I69_03160	A2I70_03175	A2I71_10410	A2I72_03065	A2I73_10075
*fbe* (*sdr*G)	Fibronectin binding protein	–	Hartford et al., 2001	–	–	–	–	–
*fmt*A	methicillin resistance protein	Tu Quoc et al., 2007	–	A2I69_04085	A2I70_04345	A2I71_09195	A2I72_04340	A2I73_09030
*fnb*	Fibronectin binding protein	Jönsson et al., 1991	–	A2I69_01875	truncated A2I70_01890 – 95	A2I71_11695	A2I72_01805	A2I73_11285
*geh*D	Lipase	–	Bowden et al., 2002	A2I69_12875	–	A2I71_00405	A2I72_01015	A2I73_02855
*ica* ADBCR	Polysaccharide intercellular adhesin (PIA)	Heilmann et al., 1996b	Cramton et al., 1999	–	–	–	–	Incomplete A2I73_00825 – 00840
*mec*A	PBP2A	Côrtes et al., 2015	Petrelli et al., 2006	–	–	–	–	–
*sdr*C,D,E	SD-repeat containing proteins	Josefsson et al., 1998	–	–	–	–	–	–
*sdr*F,G,H	SD-repeat containing proteins	–	McCrea et al., 2000	–	–	–	–	–


Two truncated genes related to biofilm formation were found in the investigated *S. xylosus* genomes. In TMW 2.1602 the *bap* gene encoding the biofilm associated protein carries a mutation. In TMW 2.1324, *fnb*, responsible for the synthesis of a fibronectin binding protein is truncated. TMW 2.1324 additionally lacks the *gehD* – lipase gene, which has been described for being involved in adhesion to collagen (Bowden et al., 2002).

### Structural Analysis of the Biofilm Associated Protein (Bap) in *S. xylosus*

**FIGURE 3:**
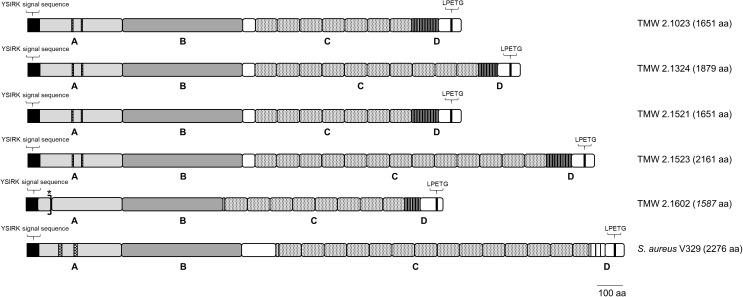
Bap structure of *S. xylosus* and *S. aureus*. The positions of the YSIRK signal sequence, the LPxTG cell wall anchor motif as well as the four domains (A–D) of the protein are shown; asterisk indicates early translation termination due to a stop codon in the aa sequence of TMW 2.1602.

In *ica*-negative strains, Bap plays a major role in biofilm formation. Thus, a detailed *in situ* structural analysis of the Bap sequence was performed (Figure 3). General structural features were adapted from Cucarella et al. (2001), and Tormo et al. (2005), and Bap structure of *S. aureus* V329 (GenBank: AY220730.1) was included into the analysis. The *bap* gene is present in the genomes of all five *S. xylosus* isolates. However, the bap sequence of strain TMW 2.1602 contains a stop codon after 94 amino acids (aa) indicating an early termination during translation. All other Bap protein sequences show typical structural characteristics. At the N-terminal site of *S. xylosus* Bap, the YSIRK signal sequence (45 aa) for extracellular secretion is followed by region A (315 aa) which contains two short repeats of 5 aa. The signal sequence is missing in the NCBI-defined open reading frame (ORF) of strains TMW 2.1023 and TMW 2.1521, however, the missing sequence is present in the unprocessed consensus sequence indicating a false delimitation of the ORF. Region B (458 aa) possesses the most conserved part of the protein as it shows the highest sequence identity among the *S. xylosus* strains (protein identity 98.7 – 100%) as well as 80% identity to the B region of *S. aureus* V329 Bap. Region C starts with a short spacer region (48 aa) followed by a long core section which encompasses a varying number of Ig-like domain repeats (83 – 86 aa). The highest number of C repeats is present in the genome of *S. xylosus* TMW 2.1523 (13), followed by TMW strains 2.1324 (10), 2.1023 (7), and 2.1521 (7). The carboxy-terminal region D is characterized by differing numbers (12 – 17) of nearly identical 6 aa tandem repeats. Additionally, it contains an LPxTG motif, which is a well-known cell wall anchor sequence in Gram-positive bacteria. Regarding Bap of TMW 2.1602, not just the early stop codon indicates a truncation of the protein, also the B region misses 73 aa, the spacer region is much shorter and the sequence of the C and D repeats is different than in the other *S. xylosus* strains, where the repeating sequence was homolog and only the amount of repeats differed among the strains. Compared to *S. aureus* V329, the biggest difference in the organization of Bap in *S. xylosus* involves the number of C and D repeats, as size and amino acid sequence differ.

## Discussion

This study investigated variations in the biofilm forming capacity of five *S. xylosus* strains isolated from raw fermented sausages in dependence of different supports and media compositions. It was demonstrated that *S. xylosus* strains with hydrophobic surface properties (TMW 2.1023, TMW 2.1324, and TMW 2.1521) adhered equally well or with minor differences to the two supports tested (hydrophobic, hydrophilic). The only hydrophilic biofilm positive *S. xylosus* strain (TMW 2.1523) on the other hand adhered distinctly better to hydrophilic than to the hydrophobic support. This is in accordance with previous studies, which have proven that bacteria with hydrophobic surface properties adhere generally well to both kinds of supports while hydrophilic strains prefer hydrophilic supports (Heilmann et al., 1996a; Planchon et al., 2006). Hydrophobic interactions are an important factor for adhesion, and cell surface hydrophobicity is influenced by a combination of the activity of autolysins such as AtlE, teichoic acids, cell surface structures, and surface net charge as well as components of the growth medium (Heilmann et al., 1997; Gross et al., 2001; An and Friedman, 2010). In this study it was further proven that biofilm formation is affected by additives to the growth medium, i.e., glucose, NaCl and lactate. The tested additives had no general stimulating or inhibitory effect on biofilm formation of all strains, but rather displayed varying strain-dependent effects. The here reported controversial effect of glucose on biofilm formation of *S. xylosus* has been reported for other staphylococci in previous studies (Hennig et al., 2007; Potter et al., 2009). For certain strains, such as *S. epidermidis* RP62A, addition of 1% glucose is essential for biofilm formation (Mack et al., 1992), which could be confirmed in this study. A generally positive effect on biofilm formation by addition of sodium chloride, previously reported for *S. epidermidis* and *S. aureus* (Rachid et al., 2000; Moretro et al., 2003) was not as distinct in the investigated *S. xylosus* strains.

Generally, the impact of glucose, sodium chloride and lactate on biofilm formation of *Staphylococcus* spp. has been mainly associated with changes in physicochemical interactions between cell and surface (Planchon et al., 2006) as well as differential expression of the *ica* operon upon stress exposure (Rachid et al., 2000; Knobloch et al., 2001). Since *S. xylosus* is *ica* negative, biofilm formation should be differently regulated by environmental stimuli. Therefore, it seems more likely that the addition of glucose or sodium chloride to the culture medium or the change of pH by lactic acid influences the physiochemical surface properties such as the surface charge of the *S. xylosus* cells (Briandet et al., 1999). These changes, can impair the cell surface hydrophobicity, change electrostatic forces between support and cell, and therefore interfere with adhesion. Quorum sensing is another regulatory factor, often discussed in context with staphylococcal biofilm formation (Vuong et al., 2003). It appears that quorum sensing effects don’t account for differences in biofilm phenotypes in this study though, as growth rates did not differ significantly in the tested media among the five *S. xylosus* strains (Data not shown). This is in contrary to the growth enhancing effects of 20 g/l NaCl addition that Planchon et al. (2006) reported. We solely observed a significantly higher growth rate and OD_max_ in TSB + 1% glucose compared to TSB lacking glucose for *S. epidermidis* RP62A (Data not shown).

Staphylococci that are *ica*-positive and thus are able to synthesize PIA often display a slime-positive phenotype on congo red agar (Petrelli et al., 2006). In this study, none of the analyzed *S. xylosus* strains showed a positive phenotype in the CRA tests, which confirmed the *in silico* analysis of *S. xylosus* being *ica* negative. It also confirms the hypothesis that *S. xylosus* TMW 2.1602 is most likely not synthesizing PIA despite carrying some genes of the *ica* operon. However, as Götz (2002) has also reported, *icaD* is of importance for PIA expression and *icaD* is missing in TMW 2.1602. TMW 2.1523 showed some characteristics of a CRA-positive phenotype by part of the colonies turning dark, rough and undulated instead of remaining round and shiny. This might be related to congo red being able to not only interact with exopolysaccharides but also proteins (Cucarella et al., 2001). Thus, either the presence of the *ess* cluster in the genome of TMW 2.1523, which mediates the excretion of certain polypeptides (Burts et al., 2008) or extracellular Bap might cause the reported phenotypic change on CRA. In general, the impact of the *ess* cluster encoded ESAT-6 secretion system on biofilm formation of *S. aureus* has been questioned in the past (Wang et al., 2016), yet for *Mycobacterium marinum* a correlation between ESAT-6 and biofilm formation has been reported (Lai et al., 2018). Therefore, the secreted polypeptides might be part of the biofilm matrix of TMW 2.1523.

To address the question of biofilm intensity formed by *S. xylosus, S. epidermidis* RP62A, known for being a strong biofilm producer, was taken into account as a reference strain in this study. Hereby, it was shown that *ica*-negative *S. xylosus* strains are able to form similar intensities of biofilm as the *ica*-positive *S. epidermidis* RP62A strain does. In order to investigate the mechanism of *S. xylosus* biofilm formation, a comparative genomic analysis of the *S. xylosus* strains was performed and genomes were screened for presence or absence of genes, which have previously been identified as being involved in biofilm formation of *S. aureus* and *S. epidermidis*. Bap seems to be a major factor in *S. xylosus* biofilm formation, as other well-known biofilm accumulation factors such as the *ica* operon and *aap* were absent in the analyzed genomes. Additionally, the physiological data support the thesis that Bap plays a major role in *S. xylosus* biofilm formation, as the biofilm negative strain TMW 2.1602 carried a truncated *bap* sequence. The importance of Bap in *ica-*negative strains has been described for other staphylococci before, e.g., Tormo et al. (2005), have proven that *ica* negative strains lose their ability to form biofilm once the *bap* gene is disrupted. It is possible though, that other, yet unknown mechanisms can contribute to biofilm formation. Comparison of the Bap sequences in *S. xylosus* demonstrated variations in the number of C and D repeats of the protein. However, it has been assumed that at least a varying number of C repeats does not influence the functionality of Bap, as for instance Cucarella et al. (2004) could not identify a correlation between number of C repeats and bap-mediated biofilm formation of *S. aureus* isolates. Furthermore, Bap has been described as being carried on the pathogenicity island SaPIbov2 in *S. aureus* (Ubeda et al., 2003). Yet, for *S. xylosus* no indicators were found that the bap locus was carried on or within a mobile genetic element.

Biofilm formation may contribute to fitness and survival of starter cultures in a particular ecological niche. This assumption is based on the principle of colonization resistance, a phenomenon well known from the human intestine where the microbiota prevents inflammation by occupying all niches along the intestinal tract (Lawley and Walker, 2013). In the sausage matrix, starters with high adhesion and biofilm forming potential may occupy microniches within the meat matrix during fermentation and thus increase their assertiveness against autochthonous staphylococci. The knowledge obtained in this study can be used to explain strain-specific differences of assertiveness in raw sausage fermentation previously identified (Vogel et al., 2017). Screening for a defined set of marker genes derived from the reported comparative genomics results may support the choice of assertive biofilm formers among *S. xylosus*. Taken together, this study demonstrated variability in biofilm formation of different *S. xylosus* strains and analyzed for the first time, which adhesion and biofilm related genes are present and absent among different *S. xylosus* strains displaying distinct phenotypes.

## Data Availability

The datasets generated for this study can be found in Genbank, CP015538, CP015539 – CP015541, CP015542 – CP015545, CP015546 – CP015551, and CP015555 – CP015556.

## Author Contributions

CS conducted all the experiments, evaluated the data, generated the figures and tables, and wrote the first draft of the manuscript. MH helped in the bioinformatics analyses. ME supervised the work of CS and helped with biofilm tests. RV initiated the project, leaded the design of the study, and supervised CS. All authors contributed to manuscript revision, read, and approved the submitted version.

## Conflict of Interest Statement

The authors declare that the research was conducted in the absence of any commercial or financial relationships that could be construed as a potential conflict of interest.

## References

[B1] An Y. H. Friedman R. J. (eds) (2010). *Handbook of Bacterial Adhesion: Principles, Methods, and Applications*. Totowa, NJ: Humana Press.

[B2] Aziz R. K. Bartels D. Best A. A. DeJongh M. Disz T. Edwards R. A. (2008). The RAST server: rapid annotations using subsystems technology. *BMC Genomics* 9:75. 10.1186/1471-2164-9-75 18261238 PMC2265698

[B3] Bose J. L. Lehman M. K. Fey P. D. Bayles K. W. (2012). Contribution of the *Staphylococcus aureus* Atl AM and GL murein hydrolase activities in cell division, autolysis, and biofilm formation. *PLoS One* 7:e42244. 10.1371/journal.pone.0042244 22860095 PMC3409170

[B4] Bowden M. G. Visai L. Longshaw C. M. Holland K. T. Speziale P. Hook M. (2002). Is the GehD lipase from *Staphylococcus epidermidis* a collagen binding adhesin? *J. Biol. Chem.* 277 43017–43023. 10.1074/jbc.M207921200 12218064

[B5] Briandet R. Meylheuc T. Maher C. Bellon-Fontaine M. N. (1999). *Listeria monocytogenes* scott a: cell surface charge, hydrophobicity, and electron donor and acceptor characteristics under different environmental growth conditions. *Appl. Environ. Microbiol.* 65 5328–5333. 10583984 10.1128/aem.65.12.5328-5333.1999 PMC91724

[B6] Burts M. L. DeDent A. C. Missiakas D. M. (2008). EsaC substrate for the ESAT-6 secretion pathway and its role in persistent infections of *Staphylococcus aureus*. *Mol. Microbiol.* 69 736–746. 10.1111/j.1365-2958.2008.06324.x 18554323 PMC2597432

[B7] Carneiro C. R. W. Postol E. Nomizo R. Reis L. F. L. Brentani R. R. (2004). Identification of enolase as a laminin-binding protein on the surface of *Staphylococcus aureus*. *Microbes Infect.* 6 604–608. 10.1016/j.micinf.2004.02.003 15158195

[B8] Chin C.-S. Alexander D. H. Marks P. Klammer A. A. Drake J. Heiner C. (2013). Nonhybrid, finished microbial genome assemblies from long-read SMRT sequencing data. *Nat. Methods* 10 563–569. 10.1038/nmeth.2474 23644548

[B9] Christensen G. D. Simpson W. A. Younger J. J. Baddour L. M. Barrett F. F. Melton D. M. (1985). Adherence of coagulase-negative staphylococci to plastic tissue culture plates: a quantitative model for the adherence of staphylococci to medical devices. *J. Clin. Microbiol.* 22 996–1006. 3905855 10.1128/jcm.22.6.996-1006.1985 PMC271866

[B10] Clarke S. R. Harris L. G. Richards R. G. Foster S. J. (2002). Analysis of Ebh, a 1.1-megadalton cell wall-associated fibronectin-binding protein of *Staphylococcus aureus*. *Infect. Immun.* 70 6680–6687. 12438342 10.1128/IAI.70.12.6680-6687.2002 PMC133066

[B11] Conlon K. M. Humphreys H. O’Gara J. P. (2002). icaR encodes a transcriptional repressor involved in environmental regulation of ica operon expression and biofilm formation in *Staphylococcus epidermidis*. *J. Bacteriol.* 184 4400–4408. 12142410 10.1128/JB.184.16.4400-4408.2002 PMC135245

[B12] Corrigan R. M. Rigby D. Handley P. Foster T. J. (2007). The role of *Staphylococcus aureus* surface protein SasG in adherence and biofilm formation. *Microbiology* 153 2435–2446. 10.1099/mic.0.2007/006676-0 17660408

[B13] Côrtes M. F. Beltrame C. O. Ramundo M. S. Ferreira F. A. Figueiredo A. M. S. (2015). The influence of different factors including *fnb*A and *mec*A expression on biofilm formed by MRSA clinical isolates with different genetic backgrounds. *Int. J. Med. Microbiol.* 305 140–147. 10.1016/j.ijmm.2014.11.011 25547264

[B14] Cramton S. E. Gerke C. Schnell N. F. Nichols W. W. Götz F. (1999). The intercellular adhesion (*ica*) locus is present in *Staphylococcus aureus* and is required for biofilm formation. *Infect. Immun.* 67 5427–5433. 10496925 10.1128/iai.67.10.5427-5433.1999 PMC96900

[B15] Cucarella C. Solano C. Valle J. Amorena B. Lasa I. Penadés J. R. (2001). Bap, a *Staphylococcus aureus* surface protein involved in biofilm formation. *J. Bacteriol.* 183 2888–2896. 10.1128/JB.183.9.2888-2896.2001 11292810 PMC99507

[B16] Cucarella C. Tormo M. A. Ubeda C. Trotonda M. P. Monzon M. Peris C. (2004). Role of biofilm-associated protein Bap in the pathogenesis of bovine *Staphylococcus aureus*. *Infect. Immun.* 72 2177–2185. 10.1128/IAI.72.4.2177-2185.2004 15039341 PMC375157

[B17] Downer R. Roche F. Park P. W. Mecham R. P. Foster T. J. (2002). The elastin-binding protein of *Staphylococcus aureus* (EbpS) is expressed at the cell surface as an integral membrane protein and not as a cell wall-associated protein. *J. Biol. Chem.* 277 243–250. 10.1074/jbc.M107621200 11684686

[B18] Fey P. D. Olson M. E. (2010). Current concepts in biofilm formation of *Staphylococcus epidermidis*. *Future Microbiol.* 5 917–933. 10.2217/fmb.10.56 20521936 PMC2903046

[B19] Flemming H. C. Wingender J. (2010). The biofilm matrix. *Nat. Rev. Microbiol.* 8 623–633. 10.1038/nrmicro2415 20676145

[B20] Freeman D. J. Falkiner F. R. Keane C. T. (1989). New method for detecting slime production by coagulase negative staphylococci. *J. Clin. Pathol.* 42 872–874. 2475530 10.1136/jcp.42.8.872 PMC1142068

[B21] Goris J. Konstantinidis K. T. Klappenbach J. A. Coenye T. Vandamme P. Tiedje J. M. (2007). DNA-DNA hybridization values and their relationship to whole-genome sequence similarities. *Int. J. Syst. Evol. Microbiol.* 57 81–91. 10.1099/ijs.0.64483-0 17220447

[B22] Götz F. (2002). Staphylococcus and biofilms. *Mol. Microbiol.* 43 1367–1378. 10.1046/j.1365-2958.2002.02827.x 11952892

[B23] Götz F. Zabielski J. Philipson L. Lindberg M. (1983). DNA homology between the arsenate resistance plasmid pSX267 from *Staphylococcus xylosus* and the penicillinase plasmid pI258 from *Staphylococcus aureus*. *Plasmid* 9 126–137. 10.1016/0147-619X(83)90015-X 6602348

[B24] Gross M. Cramton S. E. Götz F. Peschel A. (2001). Key role of teichoic acid net charge in *Staphylococcus aureus* colonization of artificial surfaces. *Infect. Immun.* 69 3423–3426. 10.1128/IAI.69.5.3423-3426.2001 11292767 PMC98303

[B25] Hartford O. O’Brien L. Schofield K. Wells J. Foster T. J. (2001). The Fbe (SdrG) protein of *Staphylococcus epidermidis* HB promotes bacterial adherence to fibrinogen. *Microbiology* 147 2545–2552. 10.1099/00221287-147-9-2545 11535794

[B26] Heilmann C. Gerke C. Perdreau-Remington F. Götz F. (1996a). Characterization of Tn917 insertion mutants of *Staphylococcus epidermidis* affected in biofilm formation. *Infect. Immun.* 64 277–282. 8557351 10.1128/iai.64.1.277-282.1996 PMC173756

[B27] Heilmann C. Schweitzer O. Gerke C. Vanittanakom N. Mack D. Götz F. (1996b). Molecular basis of intercellular adhesion in the biofilm-forming *Staphylococcus epidermidis*. *Mol. Microbiol.* 20 1083–1091. 10.1111/j.1365-2958.1996.tb02548.x 8809760

[B28] Heilmann C. Hussain M. Peters G. Götz F. (1997). Evidence for autolysin-mediated primary attachment of *Staphylococcus epidermidis* to a polystyrene surface. *Mol. Microbiol.* 24 1013–1024. 10.1046/j.1365-2958.1997.4101774.x 9220008

[B29] Hennig S. Nyunt Wai S. Ziebuhr W. (2007). Spontaneous switch to PIA-independent biofilm formation in an *ica*-positive *Staphylococcus epidermidis* isolate. *Int. J. Med. Microbiol.* 297 117–122. 10.1016/j.ijmm.2006.12.001 17292669

[B30] Hong J. Roh E. (2018). Complete genome sequence of biofilm-producing strain *Staphylococcus xylosus* S170. *Korean J. Micobiol.* 54 167–168. 10.7845/kjm.2018.54.3.308

[B31] Jönsson K. McDevitt D. McGavin M. H. Patti J. M. Höök M. (1995). *Staphylococcus aureus* expresses a major histocompatibility complex class II analog. *J. Biol. Chem.* 270 21457–21460. 7545162 10.1074/jbc.270.37.21457

[B32] Jönsson K. Signas C. Müller H.-P. Lindberg M. (1991). Two different genes encode fibronectin binding proteins in *Staphylococcus aureus*. The complete nucleotide sequence and characterization of the second gene. *Eur. J. Biochem.* 202 1041–1048. 10.1111/j.1432-1033.1991.tb16468.x 1837266

[B33] Josefsson E. McCrea K. W. Ní Eidhin D. O’Connell D. Cox J. Höök M. (1998). Three new members of the serine-aspartate repeat protein multigene family of *Staphylococcus aureus*. *Microbiology* 144(Pt 12), 3387–3395. 10.1099/00221287-144-12-3387 9884231

[B34] Knobloch J. K. Bartscht K. Sabottke A. Rohde H. Feucht H. H. Mack D. (2001). Biofilm formation by *Staphylococcus epidermidis* depends on functional RsbU, an activator of the sigB operon: differential activation mechanisms due to ethanol and salt stress. *J. Bacteriol.* 183 2624–2633. 10.1128/JB.183.8.2624-2633.2001 11274123 PMC95180

[B35] Knobloch J. K.-M. Horstkotte M. A. Rohde H. Mack D. (2002). Evaluation of different detection methods of biofilm formation in *Staphylococcus aureus*. *Med. Microbiol. Immunol.* 191 101–106. 10.1007/s00430-002-0124-3 12410349

[B36] Labrie S. J. El Haddad L. Tremblay D. M. Plante P.-L. Wasserscheid J. Dumaresq J. (2014). First complete genome sequence of *Staphylococcus xylosus*, a meat starter culture and a host to propagate *Staphylococcus aureus* phages. *Genome Announc.* 2:e0671-14. 10.1128/genomeA.00671-14 25013142 PMC4110768

[B37] Lai L.-Y. Lin T.-L. Chen Y.-Y. Hsieh P.-F. Wang J.-T. (2018). Role of the *Mycobacterium marinum* ESX-1 secretion system in sliding motility and biofilm formation. *Front. Microbiol.* 9:1160. 10.3389/fmicb.2018.01160 29899738 PMC5988883

[B38] Latasa C. Solano C. Penadés J. R. Lasa I. (2006). Biofilm-associated proteins. *C. R. Biol.* 329 849–857. 10.1016/j.crvi.2006.07.008 17067927

[B39] Lawley T. D. Walker A. W. (2013). Intestinal colonization resistance. *Immunology* 138 1–11. 10.1111/j.1365-2567.2012.03616.x 23240815 PMC3533696

[B40] Ma A. P. Y. Jiang J. Tun H. M. Mauroo N. F. Yuen C. S. Leung F. C.-C. (2014). Complete genome sequence of *Staphylococcus xylosus* HKUOPL8, a potential opportunistic pathogen of mammals. *Genome Announc.* 2:e0653-14. 10.1128/genomeA.00653-14 25059860 PMC4110218

[B41] Mack D. Siemssen N. Laufs R. (1992). Parallel induction by glucose of adherence and a polysaccharide antigen specific for plastic-adherent *Staphylococcus epidermidis*: evidence for functional relation to intercellular adhesion. *Infect. Immun.* 60 2048–2057. 1314224 10.1128/iai.60.5.2048-2057.1992 PMC257114

[B42] McCrea K. W. Hartford O. Davis S. Eidhin D. N. Lina G. Speziale P. (2000). The serine-aspartate repeat (Sdr) protein family in *Staphylococcus epidermidis*. *Microbiology* 146(Pt 7), 1535–1546. 10.1099/00221287-146-7-1535 10878118

[B43] McDevitt D. Francois P. Vaudaux P. Foster T. J. (1994). Molecular characterization of the clumping factor (fibrinogen receptor) of *Staphylococcus aureus*. *Mol. Microbiol.* 11 237–248. 8170386 10.1111/j.1365-2958.1994.tb00304.x

[B44] Moretro T. Hermansen L. Holck A. L. Sidhu M. S. Rudi K. Langsrud S. (2003). Biofilm formation and the presence of the intercellular adhesion locus *ica* among staphylococci from food and food processing environments. *Appl. Environ. Microbiol.* 69 5648–5655. 10.1128/AEM.69.9.5648-5655.2003 12957956 PMC194930

[B45] Ní Eidhin D. Perkins S. Francois P. Vaudaux P. Höök M. Foster T. J. (1998). Clumping factor B (ClfB), a new surface-located fibrinogen-binding adhesin of *Staphylococcus aureus*. *Mol. Microbiol.* 30 245–257. 10.1046/j.1365-2958.1998.01050.x 9791170

[B46] Otto M. (2008). Staphylococcal biofilms. *Curr. Top. Microbiol. Immunol.* 322 207–228. 18453278 10.1007/978-3-540-75418-3_10 PMC2777538

[B47] Palma M. Haggar A. Flock J. I. (1999). Adherence of *Staphylococcus aureus* is enhanced by an endogenous secreted protein with broad binding activity. *J. Bacteriol.* 181 2840–2845. 10217776 10.1128/jb.181.9.2840-2845.1999 PMC93727

[B48] Palma M. Wade D. Flock M. Flock J. I. (1998). Multiple binding sites in the interaction between an extracellular fibrinogen-binding protein from *Staphylococcus aureus* and fibrinogen. *J. Biol. Chem.* 273 13177–13181. 9582359 10.1074/jbc.273.21.13177

[B49] Patti J. M. Jonsson H. Guss B. Switalski L. M. Wiberg K. Lindberg M. (1992). Molecular characterization and expression of a gene encoding a *Staphylococcus aureus* collagen adhesin. *J. Biol. Chem.* 267 4766–4772. 1311320

[B50] Petrelli D. Zampaloni C. D’Ercole S. Prenna M. Ballarini P. Ripa S. (2006). Analysis of different genetic traits and their association with biofilm formation in *Staphylococcus epidermidis* isolates from central venous catheter infections. *Eur. J. Clin. Microbiol. Infect. Dis.* 25 773–781. 10.1007/s10096-006-0226-8 17089093

[B51] Planchon S. Gaillard-Martinie B. Dordet-Frisoni E. Bellon-Fontaine M. N. Leroy S. Labadie J. (2006). Formation of biofilm by *Staphylococcus xylosus*. *Int. J. Food Microbiol.* 109 88–96. 10.1016/j.ijfoodmicro.2006.01.016 16503066

[B52] Potter A. Ceotto H. Giambiagi-Demarval M. Dos Santos K. R. N. Nes I. F. Bastos Mdo C. (2009). The gene *bap*, involved in biofilm production, is present in *Staphylococcus* spp. Strains from nosocomial infections. *J. Microbiol.* 47 319–326. 10.1007/s12275-009-0008-y 19557349

[B53] Rachid S. Ohlsen K. Wallner U. Hacker J. Hecker M. Ziebuhr W. (2000). Alternative transcription factor sigma B is involved in regulation of biofilm expression in a *Staphylococcus aureus* mucosal isolate. *J. Bacteriol.* 182 6824–6826. 10.1128/JB.182.23.6824-6826.2000 11073930 PMC111428

[B54] Richter M. Rosselló-Móra R. Oliver Glöckner F. Peplies J. (2016). JSpeciesWS: a web server for prokaryotic species circumscription based on pairwise genome comparison. *Bioinformatics* 32 929–931. 10.1093/bioinformatics/btv681 26576653 PMC5939971

[B55] Rosenberg M. (2006). Microbial adhesion to hydrocarbons: twenty-five years of doing MATH. *FEMS Microbiol. Lett.* 262 129–134. 10.1111/j.1574-6968.2006.00291.x 16923066

[B56] Schaeffer C. R. Woods K. M. Longo G. M. Kiedrowski M. R. Paharik A. E. Büttner H. (2015). Accumulation-associated protein enhances *Staphylococcus epidermidis* biofilm formation under dynamic conditions and is required for infection in a rat catheter model. *Infect. Immun.* 83 214–226. 10.1128/IAI.02177-14 25332125 PMC4288872

[B57] Shankar V. Baghdayan A. S. Huycke M. M. Lindahl G. Gilmore M. S. (1999). Infection-derived *Enterococcus faecalis* strains are enriched in *esp*, a gene encoding a novel surface protein. *Infect. Immun.* 67 193–200. 9864215 10.1128/iai.67.1.193-200.1999 PMC96296

[B58] Tatusova T. DiCuccio M. Badretdin A. Chetvernin V. Nawrocki E. P. Zaslavsky L. (2016). NCBI prokaryotic genome annotation pipeline. *Nucleic Acids Res.* 44 6614–6624. 10.1093/nar/gkw569 27342282 PMC5001611

[B59] Tormo M. A. Knecht E. Götz F. Lasa I. Penades J. R. (2005). Bap-dependent biofilm formation by pathogenic species of *Staphylococcus*: evidence of horizontal gene transfer? *Microbiology* 151 2465–2475. 10.1099/mic.0.27865-0 16000737

[B60] Tu Quoc P. H. Genevaux P. Pajunen M. Savilahti H. Georgopoulos C. Schrenzel J. (2007). Isolation and characterization of biofilm formation-defective mutants of *Staphylococcus aureus*. *Infect. Immun.* 75 1079–1088. 10.1128/IAI.01143-06 17158901 PMC1828571

[B61] Ubeda C. Tormo-Mas M. A. Cucarella C. Trotonda P. Foster T. J. Lasa I. (2003). Sip, an integrase protein with excision, circularization and integration activities, defines a new family of mobile *Staphylococcus aureus* pathogenicity islands. *Mol. Microbiol.* 49 193–210. 10.1046/j.1365-2958.2003.03577.x 12823821

[B62] Vogel R. F. Lechner A. Ruhland K. Ehrmann M. A. (2017). “Assertiveness of *Staphylococcus carnosus* and *Staphylococcus xylosus* in sausage fermentation,” in *Proceedings of the 3rd International Symposium on Fermented Meats* (France: Clermont-Ferrand), 15.

[B63] Vos P. de Garrity G. M. Jones D. (eds) (2009). *Bergey’s Manual of Systematic Bacteriology: Volume 3: The Firmicutes*. Dordrecht: Springer.

[B64] Vuong C. Gerke C. Somerville G. A. Fischer E. R. Otto M. (2003). Quorum-sensing control of biofilm factors in *Staphylococcus epidermidis*. *J. Infect. Dis.* 188 706–718. 10.1086/377239 12934187

[B65] Wang Y. Hu M. Liu Q. Qin J. Dai Y. He L. (2016). Role of the ESAT-6 secretion system in virulence of the emerging community-associated *Staphylococcus aureus* lineage ST398. *Sci. Rep.* 6:25163. 10.1038/srep25163 27112266 PMC4844983

[B66] Williams R. J. Henderson B. Sharp L. J. Nair S. P. (2002). Identification of a fibronectin-binding protein from *Staphylococcus epidermidis*. *Infect. Immun.* 70 6805–6810. 10.1128/IAI.70.12.6805-6810.2002 12438356 PMC133053

[B67] Xu C.-G. Yang Y.-B. Zhou Y.-H. Hao M.-Q. Ren Y.-Z. Wang X.-T. (2017). Comparative proteomic analysis provides insight into the key proteins as possible targets involved in aspirin inhibiting biofilm formation of *Staphylococcus xylosus*. *Front. Pharmacol.* 8:543. 10.3389/fphar.2017.00543 28871227 PMC5566577

