# Supplementary figures and images for: Bap and Cell Surface Hydrophobicity Are Important Factors in Staphylococcus xylosus Biofilm Formation
Source: Front Microbiol. 2019 Jun 25;10:1387. doi: 10.3389/fmicb.2019.01387 (PMC6603148; doi:10.3389/fmicb.2019.01387)

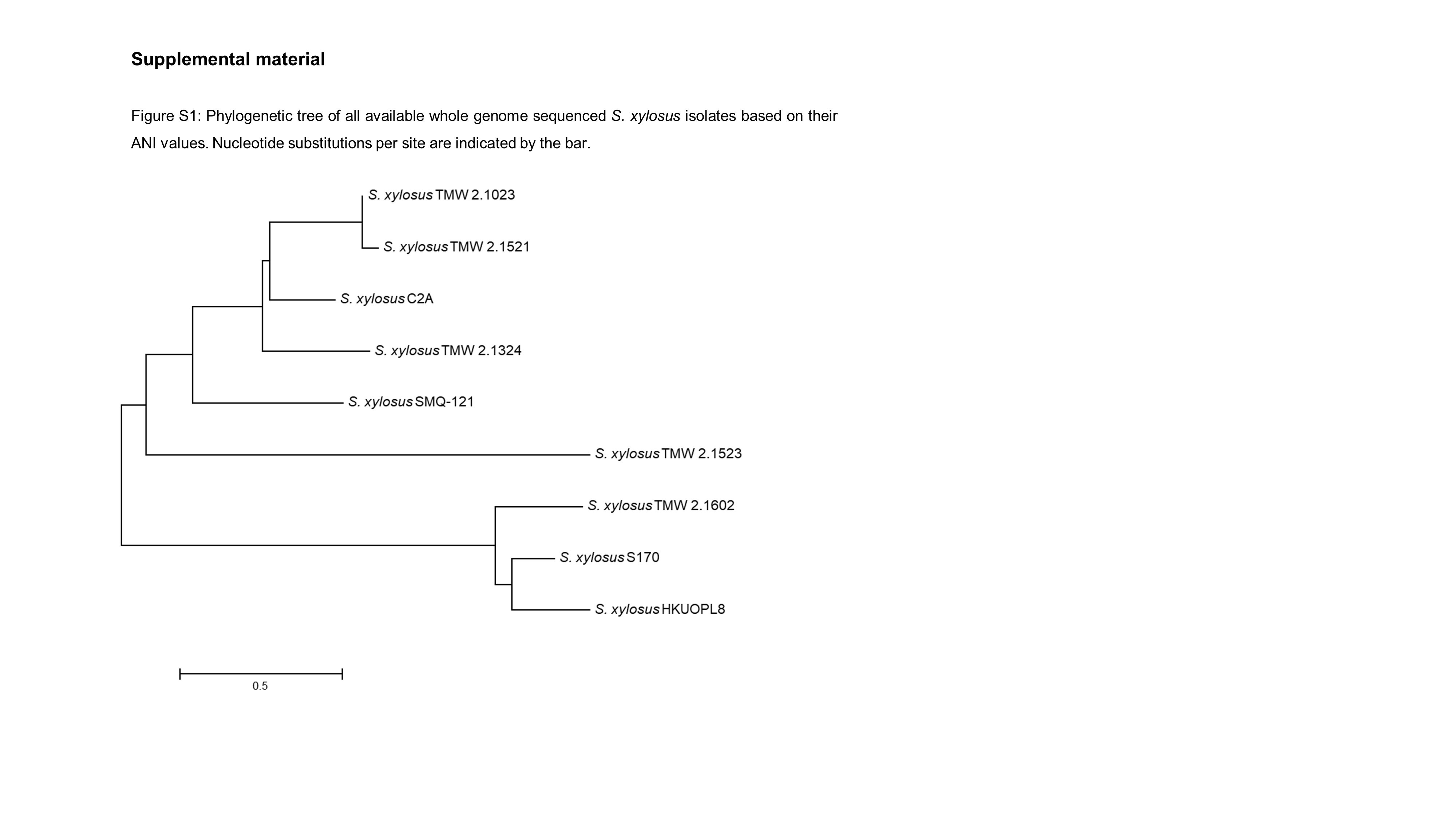

Supplement: Supplementary file 1 [file Image_1.tif]
