# Supplementary material for: Bap and Cell Surface Hydrophobicity Are Important Factors in Staphylococcus xylosus Biofilm Formation
Source: Front Microbiol. 2019 Jun 25;10:1387. doi: 10.3389/fmicb.2019.01387 (PMC6603148; doi:10.3389/fmicb.2019.01387)
Supplement: Supplementary file 2 [file Table_1.DOCX]

### Supplemental material

Table S1: General genome features of the chromosomes in S. xylosus

| ***S. xylosus* strain** | **TMW 2.1023** | **TMW 2.1324** | **TMW 2.1521** | **TMW 2.1523** | **TMW 2.1602** |
| --- | --- | --- | --- | --- | --- |
|  |  |  |  |  |  |
| total size [bp] | 2,800,497 | 2,967,040 | 3,006,054 | 2,987,684 | 2,853,146 |
| chromosome size [bp] | 2,800,497 | 2,897,817 | 2,890,169 | 2,851,252 | 2,812,049 |
| No. of plasmids | - | 2 | 3 | 5 | 1 |
| GC content [%] | 32.85 | 32.86 | 32.71 | 32.71 | 32.86 |
| Number of CDS | 2529 | 2695 | 2744 | 2745 | 2579 |
| tRNA | 57 | 59 | 57 | 57 | 57 |
| rRNA  (5S, 16S, 23S) | 9, 8, 8 | 11, 10, 10 | 8, 7, 7 | 9, 8, 8 | 8, 7, 7 |
| Coding density [%] | 83.4 | 85.1 | 87.0 | 87.2 | 83.3 |
| Isolation source | sausage | sausage | sausage | sausage | sausage |
| Genbank accession numbers | CP015538 | CP015539 – CP015541 | CP015542 – CP015545 | CP015546 – CP015551 | CP015555 - CP015556 |
